# Principles of organelle positioning in motile and non-motile cells

**DOI:** 10.1038/s44319-024-00135-4

**Published:** 2024-04-16

**Authors:** Janina Kroll, Jörg Renkawitz

**Affiliations:** Biomedical Center, Walter Brendel Center of Experimental Medicine, Institute of Cardiovascular Physiology and Pathophysiology, Klinikum der Universität, Ludwig Maximilians Universität München, Munich, Germany

**Keywords:** Organelle Positioning, Cytoskeletal Forces, Motile and Branched Cells, Cytoplasmic Streaming, Organelle-Cytoskeleton Interface, Cell Adhesion, Polarity & Cytoskeleton, Organelles

## Abstract

Cells are equipped with asymmetrically localised and functionally specialised components, including cytoskeletal structures and organelles. Positioning these components to specific intracellular locations in an asymmetric manner is critical for their functionality and affects processes like immune responses, tissue maintenance, muscle functionality, and neurobiology. Here, we provide an overview of strategies to actively move, position, and anchor organelles to specific locations. By conceptualizing the cytoskeletal forces and the organelle-to-cytoskeleton connectivity, we present a framework of active positioning of both membrane-enclosed and membrane-less organelles. Using this framework, we discuss how different principles of force generation and organelle anchorage are utilised by different cells, such as mesenchymal and amoeboid cells, and how the microenvironment influences the plasticity of organelle positioning. Given that motile cells face the challenge of coordinating the positioning of their content with cellular motion, we particularly focus on principles of organelle positioning during migration. In this context, we discuss novel findings on organelle positioning by anchorage-independent mechanisms and their advantages and disadvantages in motile as well as stationary cells.

## Abstract (web summary)

Positioning of organelles to specific intracellular locations is critical for their functionality. This review provides an overview of strategies to actively move and anchor organelles to specific locations and how this is coordinated with cellular motion.

## Graphical abstract

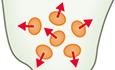

## Introduction

Eukaryotic cells possess a set of membrane-enclosed organelles such as mitochondria, lysosomes, the Golgi, the endoplasmic reticulum, and the nucleus. Although their intracellular localisation is sometimes depicted in a simplified and random manner in textbooks and review articles, advancements in the last decade have revealed that many membrane-enclosed organelles are positioned at specific intracellular sites through active mechanisms (Keys et al, 2024; Garde et al, 2022; Zheng et al, 2022; Roman et al, 2021; Moore et al, 2021; Gundersen and Worman, 2013). Similarly, increasing evidence suggests that also organelles without an enclosing membrane, such as the centrosome (Jimenez et al, 2021) or biomolecular condensates (Liao et al, 2019; Alberti and Hyman, 2021), can be positioned in a non-random manner within cells.

This asymmetric organelle distribution is not merely a passive outcome of the cytoskeletal force distribution but can be actively employed by cells to target organelles to specific intracellular locations, enabling local functions using their compartmentalised and specialised molecular content. Examples of functional organelle positioning include diverse biological processes such as non-motile muscle cells that precisely position and space their nuclei within the muscle cell syncytium (Roman et al, 2021; Azevedo and Baylies, 2020; Roman and Gomes, 2018; Gache et al, 2017; Metzger et al, 2012), proliferating neuroepithelia and radial glia cells that move their nuclei towards an apical position for an apically-localised cell division in a process termed ‘interkinetic nuclear migration’ (Strzyz et al, 2015; Taverna and Huttner, 2010; Norden et al, 2009), and motile *C.elegans* anchor cells that position their mitochondria to the cell front for local ATP production during translocation through the basement membrane (Garde et al, 2022; Kelley et al, 2019). Moreover, motile cells actively position the membrane-less centrosome to utilise it as a steering organelle (Kopf et al, 2020) and to utilise it as a distribution platform for localised secretion of extracellular proteases to facilitate the opening of tight pores in the extracellular matrix (ECM) (Infante et al, 2018).

In this review, we provide a conceptual framework of how different organelles are actively positioned in diverse cell types and biological processes. In particular, we focus on our current understanding of the functions, principles, and molecular mechanisms of organelle positioning in both stationary and motile cells. We discuss the forces that act onto organelles during their movement, including forces from actin polymerisation, myosin contractility, microtubules and their motors, the cell’s cortex, as well as intracellular flows and pressure gradients. Further, we discuss the emerging concept of anchorage-independent mechanisms for organelle positioning, such as intracellular flows, which may be particularly beneficial for highly dynamic cell types such as fast-migrating cells. While our current knowledge of organelle positioning in motile cells often derives from research on the nucleus and the centrosome, we also discuss the positioning of other organelles such as mitochondria, lysosomes, and the Golgi. We particularly emphasise mechanisms in mammalian cells, including knowledge from fibroblasts, neurons, muscle cells, immune cells, and cancer cells, while also incorporating principles from *C. elegans* nematode worms, Xenopus amphibians, intracellular pathogens, as well as single-cell eukaryotes such as the amoebae *Dictyostelium discoideum* and baker’s yeast *Saccharomyces cerevisiae*.

## Asymmetry in organelle positioning

In this section, we provide an overview of organelle positioning and emphasise its relevance for cellular and organismal functionality. We provide examples from highly ramified and extended cells like neurons, as well as highly motile cells like immune cells, to discuss the principles of active organelle positioning, as these cells constantly face the challenge of positioning their organelles either within large or highly dynamic cell bodies.

### Organelle positioning

Most cells exhibit an asymmetric organisation and thus possess a polarity, such as front-back polarity in motile cells or apical-basal polarity in epithelial cells (Bornens, 2008). This cellular polarity is particularly pronounced in specialised epithelial cells that line organs, like in the intestine or the lung epithelium, where microvilli or cilia form a highly specialised apical cellular side (Rodriguez-Boulan and Macara, 2014). In these cells, the overall polarity remains highly stable and can be maintained throughout the cell’s lifetime. However, many other differentiated cells also display polarity, resulting in an asymmetric distribution of intracellular components. This asymmetry often arises from the actin and microtubule cytoskeleton, as actin and tubulin monomers themselves exhibit an asymmetric protein structure (Li and Gundersen, 2008). Upon polymerisation, these monomers form polymeric filaments with functionally distinct ends that have distinct assembly and disassembly rates. By nucleating these asymmetric filaments at specific intracellular sites, cells generate forces with specific directionality to move cargo or membranes.

**Figure 1:**
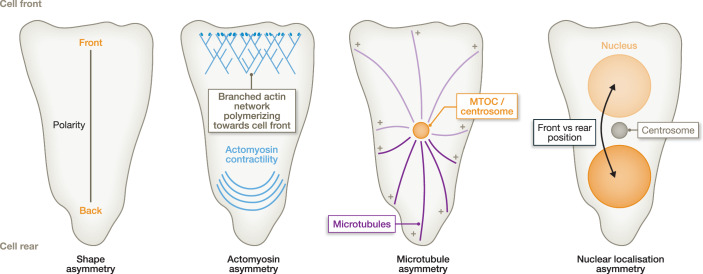
Asymmetry in motile cells. Motile cells have a distinct front-back polarity, in which the cell front (such as protrusions or lamellipodia) comprises an actin network (blue) that pushes against the cell membrane to achieve forward movement of the entire cell, and a cell rear that often contains high myosin contractility. As in non-motile cells, the centrosome/MTOC (orange) typically locates in the cell centre, providing a reference for the positioning of other organelles like the nucleus. In mesenchymal cells, the nucleus typically locates at the rear of the centrosome, whereas the nucleus typically locates at the front of the centrosome in fast amoeboid migrating cells.

Given the connectivity of many organelles to the cytoskeleton (Gurel et al, 2014), it is not surprising that the asymmetry of the actin and microtubule cytoskeleton causes an asymmetric distribution of organelles. In many cells, the membrane-less centrosome provides a reference for the relative organelle location within a cell, as it often locates at the centre of a cell due to the specific geometry of the actomyosin network (Fig. 1; see also ‘Centreing forces’) (Bornens, 2008). As the centrosome functions as a microtubule-organising centre, it orchestrates the microtubule cytoskeleton, which itself provides tracks for microtubule-dependent motor proteins that move and anchor organelle cargos like mitochondria, lysosomes, and the Golgi (see below). Consequently, minus-directed transport along microtubule tracks towards the centrosome by dynein motors causes colocalisation with the centrosome, such as observed for the membrane-surrounded Golgi (Thyberg and Moskalewski, 1999) and lysosomes (Cabukusta and Neefjes, 2018). While the nucleus is also often depicted centrally in textbooks, it is often actively moved to specific intracellular locations by anchorage-dependent and -independent mechanisms, depending on the cellular function and cell type (Gundersen and Worman, 2013). The endoplasmic reticulum (ER) is another large—or even the largest—membrane-surrounded organelle, which typically distributes throughout the entire cytoplasm with complex morphology while being directly connected to the outer nuclear membrane and the cytoskeleton (Nixon-Abell et al, 2016; Westrate et al, 2015). Yet, as we discuss below, also the ER is asymmetrically and actively positioned in different cell types.

### Challenges to position organelles in long and branched cells

Organelle positioning to distinct subcellular locations is particularly evident but also challenging in very long or highly ramified cell types like neurons, neuroepithelia, and some immune cells. A highly extended cell type is neuroepithelial cells, which are very long and thin cells with a defined apical-basal polarity. During cell division of these cells, the nucleus moves towards the apical side, where also the centrosome is located, to undergo mitosis (Taverna and Huttner, 2010). Research on this type of movement of the nucleus, also termed interkinetic nuclear movement, provided multiple important concepts, which we will discuss below, of how cells solve the challenge of moving organelles within very long cells embedded in the tissue microenvironment.

In addition to being very long, some cell types, like neurons, have a highly ramified cell morphology, generating additional challenges for organelle positioning. For instance, Golgi outposts in neurons can localise far distantly from the cell body, where the main Golgi ribbon is located (Valenzuela et al, 2020; Ori-McKenney et al, 2012). As the Golgi can act as a non-centrosomal microtubule-organising centre (MTOC) (Chabin-Brion et al, 2001), these Golgi outposts act as non-centrosomal MTOCs (Akhmanova and Kapitein, 2022). Thereby, they enable microtubule nucleation at cellular sites distant to the location of the centrosome, providing microtubule tracks for long-distance cargo delivery in these long and branched cells. Interestingly, the centrosome itself can also leave its central position in the cell body to move into cellular branches of microglia (Möller et al, 2022), a tissue-resident phagocytic cell type in the brain that has a highly ramified cellular morphology. Centrosome migration into one of the multiple cellular branches correlates with productive phagocytosis of apoptotic neurons, suggesting that effective movement and positioning of the centrosome can support phagocytosis in large and extended cells (Möller et al, 2022). Next to the Golgi and the centrosome, also the ER can localise to specific positions in the large cytoplasm of neurons, where it is enriched at dendritic branched points, and functionally implicated in the formation of new branches (Cui-Wang et al, 2012). These examples highlight that organelles can be repositioned to fulfil functions at specific intracellular sites, a concept that we discuss in more detail below. Importantly, intracellular organelle positioning is also influenced by the properties of the microenvironment, such as the positioning of the Golgi and the centrosome (Pouthas et al, 2008), as we will discuss in the section ‘Plasticity in organelle positioning’.

**Figure 2:**
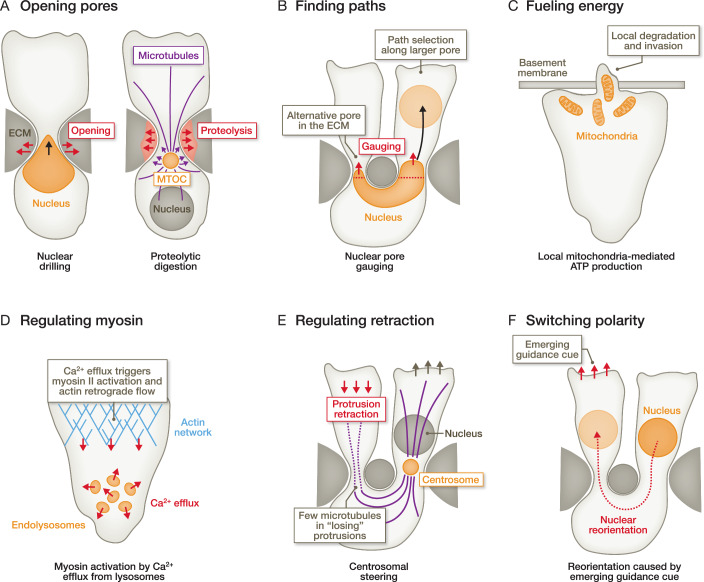
Functions of organelle positioning during cell migration. (**A**) Motile cells can employ their nucleus (orange) as a wedge or driller to open tight spaces in their local extracellular environment (ECM) in a non-proteolytic manner (left). Alternatively, cells can use the microtubule-organising centre (MTOC; which is often the centrosome) as a platform that transports vesicles loaded with proteases via microtubules (purple) to the narrow gap in the ECM, opening it in a proteolytic manner (right). Both mechanisms lead to the opening of ECM pores and thereby facilitate cellular and nuclear translocation. (**B**) To avoid translocation through narrow ECM gaps, cells can use their nucleus as a mechanical gauge, probing for the sizes of close-by pores, and thereby selecting larger pores to enable their migration along the path of least resistance. (**C**) When cells have to translocate through extremely dense and narrow ECM-like basement membranes, they can position their mitochondria close to the invasion site as a platform for local ATP production, thereby fuelling energy into the invasion process. (**D**) Ca2+ efflux from endo-lysosomes through TRPML1 channels regulates the activity of myosin II and, thus actomyosin contractility in motile cells. (**E**) Correlative data show that protrusions can turn into ‘winning’ protrusions once the centrosome localises into the protrusion, likely by stabilising the microtubule cytoskeleton within the ‘winning’ protrusion, whereas microtubule filaments in retracting protrusions without the centrosome are disassembled. (**F**) Many fast-migrating cells explore their local microenvironment with different cell fronts simultaneously. Once they decide on the cell path along the ‘winning’ protrusion, the remaining cell fronts have to be retracted, and the organelles within the retracting protrusions have to be repositioned. This mechanism allows cells to readjust their path and their organelle positioning towards emerging guidance cues.

Interestingly, some motile cells like amoeboid immune cells face a similar challenge to naturally-branched cell types: they typically extend multiple simultaneous cell fronts to explore their immediate microenvironment, leading to a complex ramified cell morphology with multiple highly dynamic cell fronts (Hadjitheodorou et al, 2021; Kopf et al, 2020; Driscoll et al, 2019; Renkawitz et al, 2019; Fritz-Laylin et al, 2017; Leithner et al, 2016; Andrew and Insall, 2007). Once a migratory path has been selected along a dominant explorative cell front, large cytoplasmic parts from the other branches (‘losing’ cell fronts) have to be retracted. This necessitates substantial repositioning of large cytoplasmic parts from the retracting cell front, which may include organelles (Fig. 2D). Indeed, long-distance repositioning events of the nucleus from ‘losing’ into ‘winning’ protrusions occur during the migration of dendritic cells, T cells, and the amoebae *Dictyostelium discoideum* (Kroll et al, 2023). This long-distance repositioning of the nucleus is efficiently mediated by actomyosin contractility, providing migrating cells the flexibility to efficiently adjust their path and intracellular content towards an emerging guidance cue, such as when chemotactic cues overrule mechanical pore size cues (Kroll et al, 2023). While many immune cells like T cells and dendritic cells migrate with an amoeboid mode that is characterised by fast velocities, low-adhesiveness to the substrate, and dynamic cell shape changes, other immune cells like macrophages (Paterson and Lämmermann, 2021) and mast cells (Kaltenbach et al, 2023) employ a more mesenchymal migration mode that is slower and characterised by stronger adhesions to the substrate (Kameritsch and Renkawitz, 2020). Nevertheless, also macrophages are highly branched cells, thereby challenging organelle positioning, a concept that remains largely unexplored in these cells. Yet, recent findings showed that macrophages receive and phagocytose dysfunctional mitochondria during the intercellular transfer from stressed cardiomyocytes, which release mitochondria within membrane-surrounded particles (Nicolás-Ávila et al, 2020).

### Challenges to position organelles in highly dynamic cells

Additional challenges for organelle positioning arise when cells transition between different states, such as during the transition between cell cycle or cell polarity phases. For instance, during cell budding of the yeast *Saccharomyces cerevisiae*, mitochondria, peroxisomes, and the ER are transported by the motor myosin V along actin cables into the bud that will develop into the new daughter cell (Knoblach and Rachubinski, 2015), enabling organelle inheritance. Furthermore, dividing mammalian cells typically acquire a round shape during mitosis (Lancaster et al, 2013), necessitating the proper distribution and positioning of organelles in the individual daughter cells (as reviewed in e.g., Carlton et al, 2020).

Independent of the cell cycle, a challenge for organelle positioning arises when cells are not stationary but motile, requiring the coordination of organelle positioning and cellular polarity along the migratory path. Directionally migrating cells establish a defined front-rear polarity, characterised by a protrusive front that generates force through actin polymerisation (Fig. 1). The protrusive front contains polymerising actin filaments, typically organised in branched actin networks nucleated by Arp2/3 (Fig. 1). Due to the high actin density in the protrusive front and the backward flow of actin towards the cell centre—which is most prominent in cells that are only low or even non-adhesive (Paluch et al, 2016; Maiuri et al, 2015; Renkawitz and Sixt, 2010; Renkawitz et al, 2009)—organelles are relatively rarely localised in the immediate protrusive cell front, especially during high-velocity movements. Yet, one can find organelles closely behind the protrusive cell front. For instance, fast-moving amoeboid cells, such as mammalian T cells, dendritic cells, and single-cell amoeba, position their nucleus directly behind the protrusive front (Renkawitz et al, 2019; Ishikawa-Ankerhold et al, 2022). This forward positioning of the nucleus is mediated by actomyosin contractility (Kroll et al, 2023) and is best described in relation to the centrosome, which typically localises to the cell centre of motile cells and behind the nucleus of fast-moving amoeboid cells (Fig. 1). In contrast, slower migrating mesenchymal cells position their nucleus behind the centrosome towards the cell rear (Gundersen and Worman, 2013; Dupin et al, 2011).

Prominent examples of rearward nuclear positioning include epithelial cells (Tsai and Meyer, 2012), astrocytes placed on micropatterns (Dupin et al, 2009), immortalised retinal pigment epithelial (RPE) cells on fibronectin-coated one-dimensional lines (Nastały et al, 2020), and motile fibroblasts moving into cell-free wounds (Luxton et al, 2010). These observations lead to the concept that more adhesive mesenchymal cells position their nucleus rearward of the centrosome, whereas low-adhesive amoeboid cells position their nucleus in front of the centrosome. It is important, however, to note that nuclear positioning may also be regulated by the microenvironment, as we will discuss below in the section ‘Plasticity in Organelle Positioning’, explaining exceptions to this rule.

Although the positioning principles of the nucleus and centrosome are increasingly well understood in various model systems, the positioning principles of other cellular organelles remain less broadly explored in motile cells. Recently, the ER in migrating fibroblasts has been shown to indirectly regulate nuclear positioning by asymmetrically decorating actin fibres when cells are moving on an adhesive surface: ventral stress fibres are decorated by the ER, preventing the coupling to the nucleus, while non-decorated dorsal actin fibres connect to the nucleus to mediate its movement (Janota et al, 2022). Notably, mitochondria are known to be moved and positioned by forces from the actin- and the microtubule cytoskeletons (López‐Doménech et al, 2018) and have recently been shown to be positioned to the protrusive cell front of *C. elegans* anchor cells that breach the basement membrane (Kelley et al, 2019). In contrast, mammalian T cells migrating on coated 2D substrates localise their mitochondria to the cell rear in a microtubule-dependent manner (Campello et al, 2006). Whether these differences in mitochondrial localisation are due to mechanistic differences in different cell types, or whether they are caused by different properties of the microenvironment remains to be investigated.

### Challenges to position organelles that exist several times

Cells face the additional challenge of positioning multiple numbers of the same organelle. This aspect is naturally the case for some organelles that exist in multiple numbers within a single cell, including mitochondria and lysosomes. Clustering of these multiple organelle numbers to a specific intracellular location can simply be achieved when they are transported by the same molecular machinery, such as for lysosome vesicles that are moved along microtubule tracks by dynein motors towards their minus ends at the MTOC (Matteoni and Kreis, 1987).

Conceptually more challenging is the proper distribution of organelles to different intracellular localisation in a spaced manner: for example, in the multinucleated muscle, nuclei are accurately and uniformly distributed and spaced within the syncytium but are enriched underneath the neuromuscular junction (Cadot et al, 2015; Ghasemizadeh et al, 2021; Roman et al, 2021), potentially facilitating the local protein translation of specialised mRNAs. Also, during exercise-induced muscle repair, nuclei move towards the damage site to deliver mRNA (Roman et al, 2021). Notably, muscle cells also contain large numbers of mitochondria. The total amount of the mitochondrial network appears to correlate with the ATP demand of the specific muscle type, and the mitochondrial morphology is influenced by the muscle architecture (Katti et al, 2022; Avellaneda et al, 2021; Mishra et al, 2015). Another challenge arises when organelles present in several numbers are prone to fusion. For example, membrane-less organelles, in particular biomolecular condensates, can fuse and thus may actively be spatially segregated to keep them apart, as has been recently shown for nuclear speckles and paraspeckles (Takakuwa et al, 2023).

In addition to these naturally occurring examples, some cell types, such as certain cancer cells (Jaiswal and Singh 2021) and occasionally dendritic cells (Weier et al, 2022), possess an amplified number of centrosomes. These multiple centrosomes typically cluster to enable bipolar cell division (Marthiens et al, 2012). Thus, when multiple numbers of the same organelle are present within a cell, additional mechanisms are needed to spatially arrange organelles by clustering, distribution, or segregation. In summary, organelles are frequently actively positioned, and we will next discuss their functional roles.

## Functional roles of asymmetric organelle positioning

Organelles can be asymmetrically distributed, but does this merely follow the asymmetry of the cytoskeleton, or does it serve specific functions at distinct intracellular locations?

### Functions of nuclear positioning in motile and non-motile cells

Substantial evidence supporting active organelle positioning for functionality derives from the discovery of specific linker proteins physically coupling organelles to the cytoskeleton, such as the LINC (linker of nucleoskeleton and cytoskeleton) complex that spans the inner and outer nuclear membrane, connecting the nucleus to the actin and microtubule cytoskeletons (Sosa et al, 2012; Starr and Han, 2002). Notably, genetic mutations in LINC complex proteins like nesprin-1, nesprin-2, and nesprin-4 have been linked to human diseases like cardiomyopathy and muscular dystrophy (Kalukula et al, 2022). Moreover, mutations in proteins responsible for moving the nucleus during interkinetic nuclear migration in the central nervous system, such as the dynein regulator Lis-1 and the microtubule-regulator doublecortin, cause lissencephaly in humans, characterised by a smooth brain surface and severe psychomotor retardation (Markus et al, 2020). These examples of nuclear mispositioning in disease underscore the functional importance of accurate nuclear positioning (Gundersen and Worman, 2013) and nuclear mechanics (Zwerger et al, 2011).

In motile cells, precise organelle positioning plays a crucial functional role in localised chemical and physical activities. When a cell migrates through a tissue microenvironment, it encounters pores in the ECM that are frequently smaller in diameter than the nucleus (Stoitzner et al, 2002; Starborg et al, 2008; Wolf et al, 2009; Weigelin et al, 2012; Kameritsch and Renkawitz, 2020). Due to the size and stiffness of the nucleus (Kalukula et al, 2022), cellular translocation through pores substantially smaller than the nucleus causes migration slowdown as the nucleus squeezes to fit through the ECM pore (Calero-Cuenca et al, 2018; McGregor et al, 2016; Thiam et al, 2016; Harada et al, 2014; Wolf et al, 2013). Thus, the nucleus acts as a bottleneck for cell migration (Wolf et al, 2013). However, the nucleus can also serve as a ‘wedge or driller’ to open ECM pores (Fig. 2A), as demonstrated in mammalian T cells translocating through an epithelial layer and *Drosophila* border cells migrating in the fly ovary (Penfield and Montell, 2022; Barzilai et al, 2017). Moreover, the positioning of the nucleus forward of the centrosome towards the protrusive front allows immune cells to gauge neighbouring pore sizes in the ECM (Renkawitz et al, 2019). This mechanical function of the nucleus enables immune cells to circumvent extremely narrow ECM pores (Fig. 2B), preventing the slowdown of motility (Renkawitz et al, 2019) and potentially reducing nuclear damage caused by extreme nuclear squeezing through pores (Denais et al, 2016; Raab et al, 2016). While these different functions of the nucleus during cell motility have been studied individually, they likely work in conjunction: when migrating cells encounter multiple ECM pores in front of them along their trafficking paths, the nucleus can act as a mechanical gauge for the different pores to choose the path of least resistance; but cells can also decide for narrow pores along strong chemotactic signals that can override the dismissive mechanical pore size cue (Kroll et al, 2023), which may be facilitated by the opening of the pore by nuclear wedging or proteolytic digestions.

### Functions of centrosome, lysosome, and mitochondria positioning in motile cells

Different types of motile cells likely employ these physical functions of nuclear positioning to varying extents: For instance, amoeboid migrating immune cells constantly patrol the tissue without generating tissue damage along their extensive trafficking paths. This is achieved by migration mechanisms, which typically do not include proteolytic digestion of the extracellular matrix, and makes pore size gauging by the nucleus an attractive strategy (Fig. 2B), which is facilitated by frontward positioning of the nucleus (Renkawitz et al, 2019). In contrast, mesenchymal cells, which can be highly proteolytically active, open ECM pores in dense microenvironments by secreting proteases that digest ECM components (Fig. 2A), such as by actin-based invadopodia enriched with ECM degrading enzymes (Monteiro et al, 2013). Notably, at least some cancer cells store the matrix metalloproteinase MT1-MMP around their centrosome and release it upon encountering narrow pores, facilitating the rearward-positioned nucleus to move through the ECM (Infante et al, 2018). Hence, the relative positioning of the centrosome is important for its function as a local protease secretion site and for the function of the nucleus as a mechanical gauge. Notably, while the positioning of the nucleus can dynamically change within a cell (see below), the centrosome typically maintains its position at the cell centre, as shown in motile fibroblasts (Gomes et al, 2005), *Dictyostelium* amoebae (Ishikawa-Ankerhold et al, 2022), and immune cells (Kroll et al, 2023). Notably, also the coordination of retraction appears to be mediated by organelle positioning: once the centrosome enters the winning protrusion of migrating dendritic cells, the remaining protrusions start to retract (Fig. 2E), mediated by myosin contractility induced by the RhoA exchange factor Lfc (Kopf et al, 2020). Together, these correlative data suggest that the positioning of the centrosome is important for its function as a steering organelle during fast amoeboid cell migration.

These examples of organelle positions in different cell types highlight the direct link between the positioning of organelles and the tasks of the respective cell type. Interestingly, in another example of cell migration through tight microenvironments, *C.elegans* anchor cells translocate through a basement membrane, using front-localised mitochondria as a local ATP production site (Fig. 2C), likely fuelling the protrusive front with ATP for extensive F-actin formation (Kelley et al, 2019; Garde et al, 2022). Thus, the asymmetric positioning of organelles can also function as a local production site of chemicals where they are most needed. Along these lines, reports identifying asymmetric positioning of RNAs towards the protrusive front of migrating cells may indicate local protein translation at subcellular sites of high protein turnover (Moriarty et al, 2022; Dermit et al, 2020; Moissoglu et al, 2020, 2019; Mardakheh et al, 2015; Mili et al, 2008). The forces driving the locomotion of motile cells are further regulated by lysosomes. Studies in amoeboid migrating dendritic cells, T cells, and *Dictyostelium* amoeba identified the release of Ca2+ from lysosomes via the channel TRPML1 (transient receptor potential cation channel, mucolipin subfamily, member 1) as a regulator of actin organisation and myosin II activity, and thus migration velocity (Bretou et al, 2017; Dehio et al, 2024).

In summary, active organelle positioning is functionally critical for chemical as well as mechanical functions in both motile and non-motile cells.

## Forces and anchors for organelle positioning

In this section, we will discuss the principles and molecular mechanisms of how organelles are moved to their specific intracellular sites to solve the above-mentioned challenges. In particular, we discuss the forces and anchoring strategies that move and position organelles.

### Cytoskeletal tracks

**Figure 3:**
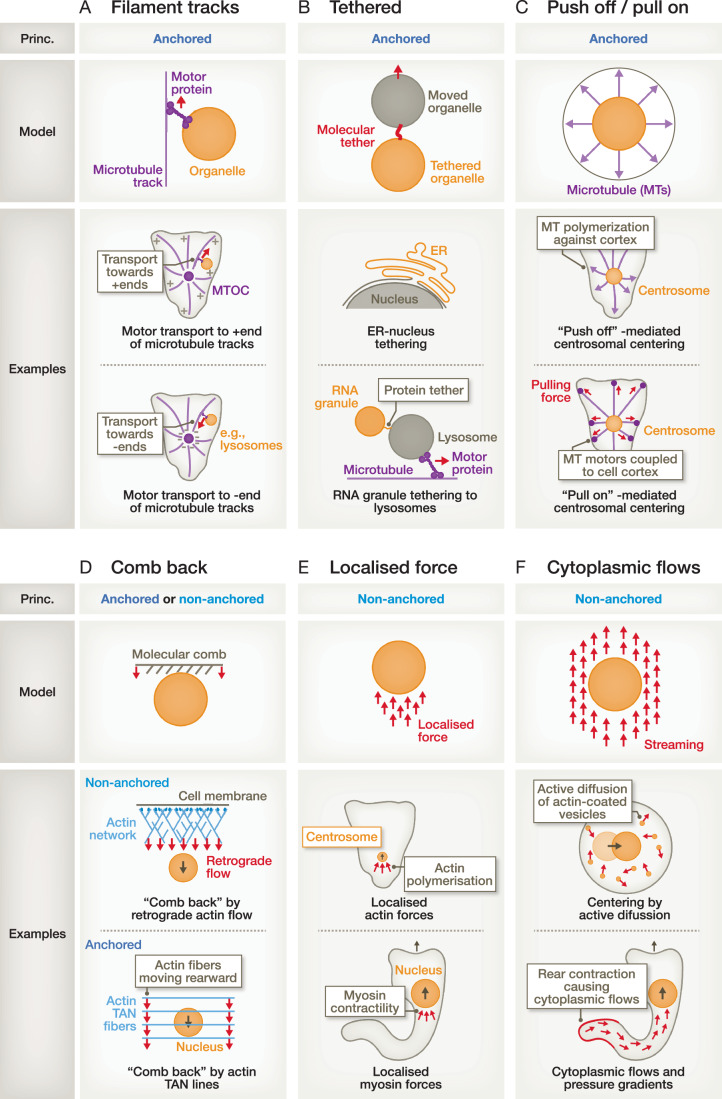
Forces and mechanisms to move and position organelles. (**A**) Microtubule filaments provide tracks for motor-based transport of organelles towards the plus- and minus-ends of microtubules. (**B**) Organelles can be indirectly moved and positioned, when they are directly connected (‘tethered’) to other organelles that move, like in the case of the direct connection between the nucleus and the ER, or by a protein tether connecting RNA granules to the lysosome. (**C**) Radially growing microtubules (MTs) can serve as a centring structure by pushing their plus ends against the cell cortex and/or by pulling them towards the cell periphery by motors that are coupled to the cell cortex. (**D**) The actin cytoskeleton can serve as a ‘molecular comb’, combing back intracellular components like organelles. This mechanism is active in fibroblasts that start to migrate in cell-free areas, in which actin fibres (called TAN lines) that are attached to the nucleus via the LINC complex, comb back the nucleus towards the cell rear. Hypothetically, also the rearward-directed retrograde flow of branched actin networks could comb back cellular organelles from the cell front towards the cell body. Notably, such a mechanism would function without directly anchoring the organelles towards the flowing actin network. (**E**) Similarly, a localised force at the back of an organelle could push the organelle forward without the need for anchoring structures, as it may likely be the case for nuclear positioning by rearward localised myosin activity or centrosome positioning by rearward localised actin polymerisation. (**F**) Generally, organelles may be moved by advection, using intracellular fluid streaming in an anchorage-independent manner, which may, for example, be caused by the contraction of the cell rear.

Many organelles are actively positioned via directly targeted forces, such as pulling and pushing forces (Marks and Petrie, 2022). Most intuitively, cytoskeletal filaments such as microtubules and actin fibres serve as tracks for their respective motor proteins, which are themselves connected via adaptor proteins to various cargos, including organelles (Fig. 3A). For instance, kinesins and dyneins move along microtubules towards their microtubule-plus and -minus ends, respectively (Hirokawa, 1998). Thereby, these motors transport and position organelle cargos, such as dynein positioning the Golgi (Yadav and Linstedt, 2011). Hence, the organisation of the microtubule cytoskeleton serves as a platform for organelle distribution and positioning (Akhmanova and Kapitein, 2022). The detailed mechanisms and functions of motor-based transport along microtubule tracks have been reviewed elsewhere, especially in the context of organelle transport within neurons (Cason and Holzbaur, 2022). Instead, we here focus on recently emerging principles of organelle positioning independent of motor-based movement along cytoskeletal tracks.

### Tethering organelles

Organelles can also be displaced by other intracellular structures. In particular, larger and stiffer organelles like the nucleus are intuitively able to physically displace other organelles, but also smaller intracellular structures like fat-filled lipid droplets can deform the nucleus (Ivanovska et al, 2023) and even push the nucleus away towards the cellular periphery (Gundersen and Worman, 2013). More actively, organelles could be indirectly moved by being connected to other, transported organelles by inter-organelle contact sites (Guo et al, 2018), such as the direct membrane connection between the ER and the nucleus (Fig. 3B) and contact sites between the ER and endosomes, lysosomes, peroxisomes, and mitochondria (Phillips and Voeltz, 2016; Raiborg et al, 2015). In epithelial sheets, cell-cell junctions provide another way to tether organelles, as revealed by the association of the ER to desmosomes, a specialised and adhesive intercellular junction structure (Bharathan et al, 2023). Notably, also the existence of contact sites between membrane-less ribonucleoprotein (RNP) granules and the membrane-surrounded ER has been recently revealed, which regulate the fission of the membrane-less granules (Lee et al, 2020). In addition to tethering mechanisms by inter-organelle contact sites, membrane-less RNA granules have recently been observed to hitchhike lysosomal transport along microtubule tracks, using the protein annexin A11 as a tether between the membrane-less RNA granule and membrane-enclosed lysosome, mediated by a lysosomal membrane binding domain and a low-complexity domain that incorporates into RNA granules (Liao et al, 2019) (Fig. 3B).

### Anchoring organelles

To couple the force that has been generated by the cytoskeleton to the organelle, classical organelle movement strategies typically involve anchoring proteins that are located on the outer surface of the organelle and interact with the cytoskeleton. Anchoring is, for example, required for nuclear movement in diverse cell types. The well-studied LINC complex mediates the anchorage of the nucleus to the cytoskeleton, being composed of SUN proteins in the inner nuclear membrane and KASH/nesprin proteins in the outer nuclear membrane that interact together and thereby bridge the perinuclear space (Janota et al, 2017; Ungricht and Kutay, 2017; Sosa et al, 2012). To couple to the cytoskeleton, KASH domain proteins interact with microtubules, actin filaments, and intermediate filaments, often mediated by adaptors, such as the microtubule motor proteins dynein and kinesin. Yet, the nucleus can also couple to the microtubule cytoskeleton in a LINC-independent manner via nuclear pore complexes (Janota et al, 2017). A prominent example of nuclear movement by anchorage to the cytoskeleton occurs in fibroblasts migrating towards the cell-free space of a wound scratch assay: the nucleus is moved towards the cell rear by actin fibres called TAN (transmembrane actin-associated nuclear) lines that move rearward and transmit the force via the LINC complex directly to the nucleus (Luxton et al, 2010). Thus, the cytoskeleton is here not employed as a railway track for motors but as a ‘comb’, combing back the nucleus towards the rear (Fig. 3D). As the rearward-moving TAN lines are anchored to the nucleus and not to the centrosome, this mechanism ensures the rearward movement of the nucleus while the centrosome maintains its relative position. Whether this TAN line-based mechanism also exists in 3D tissue microenvironments remains to be elucidated.

Fibroblasts migrating in highly confining and crosslinked ECM have been shown to employ a lobopodial migration mode (Petrie and Yamada, 2015; Petrie et al, 2014), in which fibroblasts adhere to the ECM and generate actomyosin-based pulling forces onto the nucleus, as demonstrated by the observation of a nuclear rearward drift upon local inhibition of actomyosin contractility at the cellular front (Petrie et al, 2014). Nuclear anchorage in lobopodial fibroblasts is achieved by nesprin-3 and vimentin, leading to a piston-like movement of the nucleus through the cytoplasm, pressurising the front and thereby generating bleb-like protrusions. Furthermore, it has been shown that the LINC complex protein nesprin-2 accumulates in a polarised manner at the tip of the nucleus when fibroblasts translocate through narrow pores, indicating forces from the front pulling the nucleus through narrow microenvironmental gaps (Davidson et al, 2020). Additionally, other adaptor proteins have been described, such as amphiphysin 2 (*BIN1*), which acts as a linker between the cytoskeleton and the nucleus via nesprins (Falcone et al, 2014; D’Alessandro et al, 2015).

While these examples highlight anchorage strategies of membrane-surrounded organelles, also organelles without a surrounding membrane can be anchored. The best example is the centrosome, as positioning organelles by microtubules (Schmidt and Stehbens, 2024) or their motor proteins require that microtubules are anchored to the centrosome. Thereby, the generated force does not lead to the displacement of the microtubule itself, but of the cargo. At the centrosome, microtubule minus-ends are anchored by anchoring proteins like ninein and γ-TuRC that provide linkage to the stable centriolar pair at the core of the centrosome (Akhmanova and Kapitein, 2022; Delgehyr et al, 2005; Bornens, 2002; Piel et al, 2000). Thus, anchorage does not occur at the outer surface but at the inner core of the organelle.

### Centring forces

The membrane-less centrosome often localises approximately to the cell centre. But how does it find the centre? One mechanism of centring employs the activity of the centrosome as a microtubule-organising centre (MTOC): by nucleating microtubules from the centrosome, microtubules can grow until they hit a resistance, such as the cell’s cortex or plasma membrane. Thereby, these pushing forces can centre the centrosome (Fig. 3C), provided the microtubules are sufficiently stiff to tolerate the necessary pushing force before they buckle (Wühr et al, 2009), as suggested, for example, in fission yeast (Tran et al, 2001). Alternatively, motors within the cell’s cortex could pull on microtubules, when they are attached to limited sites in the cell’s cortex, thereby centring the centrosome by pulling forces (Fig. 3C) (Grill and Hyman, 2005), a mechanism demonstrated to be active in *C. elegans* (Grill et al, 2001) and *S. cerevisiae* (Adames and Cooper, 2000). Yet, in very large cells like in Xenopus eggs, these mechanisms are unlikely to be functional as microtubules are too short to reach the cellular periphery at all cell sides (Wühr et al, 2009). However, the centrosome still finds the cell centre by mechanisms involving the actin cytoskeleton as a boundary (as we will discuss in the next paragraph) and anchorage-independent mechanisms based on intracellular dynamics and diffusion (as we will discuss in the section ‘anchorage-independent organelle positioning’). This caused substantial discussions about the mechanisms of centrosome centring, probably with different mechanisms functioning in different model systems, as reviewed previously (Deshpande and Telley, 2021).

The interaction of microtubules with other organelles, such as the nucleus, adds complexity to interpreting centrosome centreing mechanisms. Recent findings using enucleated cytoplasts attached to defined micropatterned shapes showed that the centrosome typically localises to the cell centre when the cell has a round shape. However, its position can be off-centred when the cell has an asymmetric shape and asymmetric distribution of actin, where the actin network appears to act as a boundary for microtubule growth (Jimenez et al, 2021). In vitro reconstruction with purified actin and microtubules support this mechanism, showing that an asymmetrical actin cortex leads to an asymmetrical MTOC position (Yamamoto et al, 2022). Together, these data suggest that the architecture of the actin cytoskeleton itself contributes to centrosome centreing. It will be interesting to explore such an actin-based mechanism in different cell types with different architectures and properties of the actin network, such as in lowly or non-adhesive cells like immune cells that typically do not possess thick and stable actin fibres but dynamic actin retrograde flows.

### Anchorage-independent organelle positioning

As described above, many models of organelle positioning are based on movement via anchoring linkers to the cytoskeleton. These anchoring mechanisms are also employed to immobilise the organelle to counteract diffusion. However, substantial evidence suggests that cells may employ mechanisms for organelle positioning that are independent of anchorage. In Drosophila nurse cells, actin cables polymerise from the plasma membrane towards the nucleus (Huelsmann et al, 2013), generating a pushing force that does not necessarily require anchorage to the moved object. Conceptually, the to-be-moved organelle can also be itself not anchored but carry an anchored nucleator for actin polymerisation. This principle of an anchored nucleator is employed by the intracellular pathogen *Listeria* (Lambrechts et al, 2008), in which the *Listeria* surface-anchored protein ActA directly activates the Arp2/3 complex of the host cell, generating a forward-pushing actin comet behind the pathogen (Welch et al, 1998), and the intracellular bacterium *Rickettsia* (Haglund et al, 2010), where formin- and Arp2/3-based nucleation generates the comet-like movement. Notably, this principle has recently been shown to also be employed by organelles, as mitochondria use twin comet tails, induced by an unknown nucleator, to drive their movement in a comet-like manner (Moore et al, 2021).

In addition to these mechanisms, increasing evidence suggests that organelles are also moved by advection—which is the transport of material by fluid flow (Illukkumbura et al, 2020)—using intracellular fluid streaming as an anchorage-independent movement strategy. Indeed, experiments in the 1970s showed that plasma membrane receptors accumulate at the cellular rear of migrating lymphocytes when externally crosslinked by antibodies (Taylor et al, 1971; Bray and White, 1988). This process, known as ‘antigen capping’ or ‘receptor capping’, drags crosslinked transmembrane receptors by the retrograde actin flow towards the cellular back. But do intracellular flows and diffusion also impact the positioning of organelles? Interestingly, in mouse oocytes which lack centrosomes, the positioning of the nucleus is driven by the diffusion of actin-coated vesicles that generate a propulsive force to move the nucleus to the cell centre in an anchorage-independent manner (Almonacid et al, 2015). Further, there is increasing evidence that a localised contractile activity of myosin may lead to the movement of organelles without requiring the anchorage of the organelle. In muscle cells, individual nuclei in the syncytium can be moved by actomyosin activity even when the LINC complex is not functional (Roman et al, 2017), while the accurate spacing between the individual nuclei requires the LINC complex (Roman et al, 2017). Furthermore, myosin II enriches behind the nucleus of migrating dendritic cells when the nucleus has to be repositioned from a ‘losing’ towards a ‘winning’ protrusion (Kroll et al, 2023), suggesting a mechanism in which a rearward localised contractility pushes the nucleus forward (Fig. 3E). Horizontal cells in the zebrafish retina move their nucleus with rearward localised actin activity and frequent nuclear shape changes (Amini et al, 2022). Similarly, nuclear movement in interneurons is accompanied by actin localised behind the nucleus and depends on myosin activity (Silva et al, 2018). Moreover, recent data showed that some cells, like HT1080 fibrosarcoma cells, generate pushing forces from the cell rear by contracting the cell’s rear cortex, leading to high cytosolic pressures in the back to push the nucleus through narrow gaps (Keys et al, 2024). Generally, a rearward localised contractility mechanism likely causes streaming within the cytoplasm, which cells can employ to move their organelles by advection (Fig. 3F). This mechanism will likely function particularly well on large organelle cargos within cells that are confined by the ECM, providing a large organelle surface being exposed to the streaming force and preventing the streaming force from bypassing the organelle on its sides, causing an intracellular pressure difference in the cytoplasm before and behind the organelle. This model is supported by findings in mouse oocytes, in which the diffusion of actin-coated vesicles leads to centring movement of the large nucleus and large inert oil droplets but not of smaller fluorescent particles (Colin et al, 2020).

Interestingly, the mechanisms of anchorage-independent organelle movement show similarities with the mechanisms driving the movement of amoeboid cells. Hallmarks of amoeboid cells are rearward localised myosin activity, their constantly changing cell shape, and their movement without tightly anchoring to the extracellular microenvironment (Yamada and Sixt, 2019; Paluch et al, 2016). In analogy and intracellularly, the nucleus can be moved by a rearward localised contraction, presumably without directly anchoring the nucleus to the cytoskeleton (Kroll et al, 2023; Amini et al, 2022; Keys et al, 2024; Roman et al, 2017). Such an anchorage-independent mechanism has the advantage of functioning in a fast and flexible manner, rapidly pushing the nucleus forward by rearward contractility, without the need to constantly dismantle and reassemble anchorage structures between the nucleus and the cytoskeleton. Thereby, this principle may be particularly well-suited for highly dynamic cells such as immune cells, amoeba, and some cancer cell types, where the cell shape constantly changes, and organelles like the nucleus have to be constantly moved to new intracellular positions. For instance, immune cells like dendritic cells, T cells, and the single-cell amoeba *Dictyostelium* frequently adapt their nuclear position between different alternative cell fronts when migrating in 3D microenvironments (Kroll et al, 2023). As the nucleus has to travel for long intracellular distances from a ‘losing’ towards a ‘winning’ protrusion (Fig. 2F), which is associated with two rapid switches in the nucleus-centrosome polarity (Kroll et al, 2023), the anchorage-independent nuclear movement would provide the necessary dynamics and velocity for this nuclear behaviour. Thus, it might not be surprising that neutrophil granulocytes, a white blood cell type of the innate immune system that moves in an amoeboid manner with high velocities (Nourshargh et al, 2010), have been described to be LINC-less (Olins et al, 2009), possibly suggesting a missing tight linkage between the nucleus and the cytoskeleton. Notably, anchorage-independent mechanisms for intracellular organelle positioning would likely also alter the degree of mechanical stress that is exerted onto the nucleus. Considering that gene expression is regulated by the mechanical properties of the microenvironment via the linkage of cytoskeleton forces to the nucleus (Kechagia et al, 2019), an anchorage-independent mechanism of nuclear movement may have important implications for gene expression as it may relieve mechanical stress from the nucleus and thereby reduce events of mechanically triggered nuclear envelope rupture. Nevertheless, whether there is indeed no anchorage or whether there are alternative anchorage mechanisms in fast-migrating cells has to be experimentally addressed. In particular, the proximity between the centrosome and the nucleus in amoeboid migrating cells suggests a direct linkage between these organelles, presumably via the microtubule cytoskeleton. Yet, even if the centrosome is tethered to the nucleus, the nucleus could still be moved by the above proposed anchorage-independent mechanism, and thereby would rather drag along the centrosome with its movement. Such a strategy may allow coupling between the nucleus and the centrosome to ensure that both organelles move relatively simultaneously into the cellular branch along the ‘winning’ cellular path while allowing flexible movement of the organelles without tight anchoring structures. (Lämmermann and Sixt, 2009; Moreau et al, 2020; Kameritsch and Renkawitz, 2020).

Current knowledge of anchorage-independent organelle positioning mechanisms derives from studies on nuclear positioning. Thus, investigations on this mechanism for the positioning of other organelles, including organelles of different sizes and interactions with the cytoskeleton, will characterise the utilisation of anchorage-independent mechanisms to position organelles. Generally, it is important to note that the different types of forces and anchorage strategies described in this section may also oppose each other, such as myosin forces opposing microtubule forces (Lin et al, 2016; Kapitein et al, 2013; McIntosh et al, 2015), until the final organelle localisation is established by a force equilibrium. Similarly, forces can act in parallel to each other in a complementary manner, such as for the establishment and maintenance of the large and dynamic ER network, which is moved by motors along the microtubule network, moved by coupling to growing microtubule tips, and moved by its contact to other organelles such as lysosomes (Lu et al, 2020; Zajac et al, 2013; Friedman et al, 2010; Waterman-Storer et al, 1995; Lee and Chen, 1988). Considering that anchorage-dependent mechanisms likely achieve a more precise intracellular position of an organelle than a more diffuse anchorage-independent pushing mechanism towards the cell front, it will be interesting to further observe how these two principles may act in a complementary manner to achieve organelle positioning in a fast and flexible but also robust manner.

## Plasticity in organelle positioning

Importantly, the specific positions of organelles described earlier are not necessarily fixed. They can change according to the cellular activity and the properties of the extracellular microenvironment. An example of organelle repositioning due to changes in cellular activity derives from T cells: T cells typically migrate with a nucleus-forward and centrosome-rearward configuration, but this configuration reverts when T cells engage in an immune synapse with an antigen-presenting cell or target cells, causing the centrosome to relocate towards the immune synapse (Pineau et al, 2023; Krummel and Macara, 2006). This reorientation depends on dynein, CDC42, and formins (Stowers et al, 1995; Combs et al, 2006; Gomez et al, 2007), and is required for T-cell responses and target cell killing (Kuhn and Poenie, 2002; Stinchcombe et al, 2006). Also, dendritic cells reorganise their actomyosin architecture and organelle positioning when they mature upon encounter with danger signals such as bacterial cell wall components like lipopolysaccharides (LPS). Upon maturation, the actin cytoskeleton reorganises from a preferential frontward to a rearward localisation (Vargas et al, 2016), which is regulated by the release of calcium from rearward-located lysosomes through Trpml1 channels (Bretou et al, 2017).

Apart from the dynamic repositioning of organelles for functional adaptation, properties of the microenvironment can impact the spatial positioning of organelles. This influence of the microenvironment has been demonstrated for the positioning of the centrosome, using adhesive two-dimensional (2D) patterns of different shapes (Théry et al, 2006; Hale et al, 2011). Further, investigations of Golgi positioning using adhesive one-dimensional (1D) lines, where cells typically behave similarly to 3D microenvironments but differently than on 2D surfaces, showed a preferential localisation of the Golgi behind the nucleus on 1D lines but in front of the nucleus on 2D surfaces (Pouthas et al, 2008). Moreover, imaging of mitochondria during the migration of breast cancer cells through tunnels within collagen matrices showed a more preferential localisation of mitochondria towards the cell front in more confining tunnels (Mosier et al, 2023).

Considering that microenvironmental adhesiveness and the degree of cellular confinement directly regulate the degree of myosin-mediated contractility (Lomakin et al, 2020; Liu et al, 2015; Lämmermann and Sixt, 2009), it makes intuitive sense that organelle distribution is influenced by the microenvironment. This plasticity is likely particularly important for highly motile cells that encounter various microenvironments on their trafficking routes. It is attractive to speculate that organelle movement may be largely driven by anchorage-independent mechanisms when fast-migrating cells move in loose environments. However, when they encounter denser environments or tight obstacles, they may switch to an anchorage-dependent pulling mechanism, or even use pushing and pulling by anchorage-dependent and -independent mechanisms simultaneously to generate sufficient forces to overcome tight barriers. Thus, the above-described forces are non-mutually exclusive but likely support each other to adapt to the physiological situation. Given that centrosome positioning also changes with the contractility of the cell type (Jimenez et al, 2021), the findings that the nucleus senses the degree of cellular confinement, leading to increased cellular contractility in highly confining environments (Lomakin et al, 2020; Venturini et al, 2020), also directly suggests that organelle positioning depends on the degree of cellular confinement. Together, these findings suggest that plasticity in organelle positioning caused by the microenvironment is mostly dictated by the degree of (i) organelle anchorage strength, (ii) cellular contractility, (iii) and microenvironmental confinement.

## Conclusions

We are only at the beginning of understanding the mechanisms and functions of active organelle positioning and how they are influenced by the cell type, cell state, and the tissue microenvironment (see Box 1). While the positioning of the centrosome and the nucleus are increasingly well explored, it is tempting to speculate that many more organelles, including membrane-less organelles and biomolecular condensates, are actively positioned to either provide metabolites and cellular building blocks to the subcellular localisation where they are needed, and to counteract cytoplasmic mixing during dynamic morphological changes, or to minimise their exposure to mechanical stresses. Regarding the latter, it will be important to discover how organelles shield themselves to maintain their integrity while they are exposed to the forces that move and anchor them to specific cellular sites. Moreover, studying the positioning of membrane-less organelles may uncover novel principles for movement and anchorage, given the lack of possibility to anchor membrane-less organelles to the cytoskeleton via membrane-spanning proteins. Considering that an increasing number of reports identify the importance of proper spatial organelle arrangement for cellular functions, future research may also uncover additional roles of organelle positioning in human diseases.

Next to these conceptual gaps of knowledge, research on the underexplored topic of organelle positioning may well lead to surprises in the employed molecular mechanisms, such as findings showing that different cytoskeletal protein isoforms may have different roles in organelle positioning (Roman et al, 2017) and recent findings suggesting that the LINC complex protein ANC-1 in C- elegans is not only required for nuclear positioning but also for positioning of the ER, mitochondria, and lipid droplets (Hao et al, 2021; Fischer et al, 2022). Additionally, we are only at the beginning of understanding how asymmetric patterns of posttranslational modifications of cytoskeleton components like microtubules (Lavrsen et al, 2023; Janke and Magiera, 2020) influence organelle localisation, such as the recently identified role of polyglutamylated microtubules in ER and lysosome positioning (Zheng et al, 2022). From a conceptual perspective, current research on organelle positioning often focuses on how the cytoskeleton moves and anchors organelles. Yet, how the localisation of organelles to specific sites itself impacts the organisation of the cytoskeleton is less well explored and may uncover important reciprocal relationships between cytoskeletal architecture and organelle positioning. Along these lines, research visualising multiple organelles simultaneously will unravel reciprocal relationships between their localisation.

Recent methodological advances like the optogenetic control of organelle transport (Bergeijk et al, 2015, 2016), the precise control of microenvironmental properties (Théry et al, 2006; Lautenschläger and Piel, 2013; Garcia-Arcos et al, 2019; Kroll et al, 2022), the usage of extremely long and ramified cells like neurons (Tas et al, 2017) or spatiotemporally extremely dynamic cells like immune cells (Moreau et al, 2020; Clausen et al, 2022) as cellular models, the subcellular control of cytoskeletal activity (Meiring et al, 2022; Wittmann et al, 2020; Borowiak et al, 2015; Ballister et al, 2015), and the advances in computational analysis (Schmied et al, 2023; Berg et al, 2019) have the potential to uncover fundamental principles and their underlying mechanisms in organelle positioning. Studying organelle positioning in a quantitative high throughput manner across different cell types, organisms, stimuli, microenvironmental contexts, and diseases, could also reveal quantitatively whether some organelles maintain a rather fixed position that is conserved throughout different organisms, cell types, and contexts, or may more dynamically adapt to the cellular and physiological requirement. These approaches may also lead to the characterisation of whether organelle positioning evolved with cell motility strategies and the complexity of multicellular organisms (Brunet and Booth, 2023; Fritz-Laylin, 2020) and whether cancer cells or intracellular parasites, such as the obligate intracellular parasite *Toxoplasma gondii*, hijack mechanisms of precise, efficient, and robust organelle positioning.

Box 1 In need of answers

(i) How does the microenvironment regulate organelle positioning?
(ii) Do membrane-less organelles utilise specific mechanistic principles for their active movement, positioning, and anchorage?
(iii) Do organelles shield themselves to maintain their integrity while being exposed to intracellular positioning and anchoring forces?
(iv) Do cancer cells or intracellular parasites hijack the principles of active organelle positioning to their benefit?
